# Versatile MoS_2_ Nanosheets in ITO-Free and Semi-transparent Polymer Power-generating Glass

**DOI:** 10.1038/srep12161

**Published:** 2015-07-16

**Authors:** Xiaotian Hu, Lie Chen, Licheng Tan, Yong Zhang, Lin Hu, Bing Xie, Yiwang Chen

**Affiliations:** 1 School of Materials Science and Engineering/Institute of Polymers, Nanchang University, 999 Xuefu Avenue, Nanchang 330031, China; 2 Jiangxi Provincial Key Laboratory of New Energy Chemistry, College of Chemistry, Nanchang University, 999 Xuefu Avenue, Nanchang 330031, China; 3 Center of Analysis and Testing, Nanchang University, 235 Nanjing East Road, Nanchang 330047, China

## Abstract

Chemical exfoliated ultra-thin MoS_2_ nanosheets (NSs) with well 2D structure were demonstrated for interfacial layers and Ag nanowires composite transparent electrode in polymer solar cells (PSCs). The smooth and uniform n-type and p-type (after the plasma treatment) MoS_2_ NSs could improve fill factor of devices and light absorption in active layer. The optimized Ag nanowires–MoS_2_ NSs (AgNW-MoS_2_ NSs) transparent electrode presented a low sheet resistance of 9.8 Ω sq^−1^, and the corresponding transmittance also exhibited a high value of 93.1% at 550 nm. As a result, ITO-free PSCs based on AgNW-MoS_2_ NSs/n-MoS_2_ NSs cathode and p-MoS_2_ NSs/Ag anode achieved a highest PCE of 8.72%. Furthermore, a high efficiency (6.55%), large area and low cost semi-transparent power-generating glass was obtained, after reducing the thickness of top Ag electrode from 100 nm to 30 nm. To our best knowledge, it is the highest performance for semi-transparent PSCs devices reported up to now. The novel semi-transparent power-generating glass showed good performance and color purity for commercial applications in the near future.

Polymer solar cells (PSCs) are extremely attractive candidates for use in next-generation solar cell technologies because of their mechanical flexibility, light weight, and cost-effective production through solution-based manufacturing processes^1^,^2^,^3^,^4^,^5^,^6^,^7^,^8^. To reach commercialization and mass production, PSCs must exhibit high performance and special applications^9^,^10^,^11^.

A typical PSC is based on the bulk-heterojunction (BHJ) device configuration, which sandwiches a layer of polymer donor and fullerene acceptor blend between a transparent electrode (such as indium tin oxide (ITO)) and an opaque, reflective back electrode (such as aluminum (Al) or argentum (Ag))^12^. There are also interfacial layers existing between active layer and electrodes, and the most common anode interfacial layer is poly (3, 4-ethylene dioxythiophene): poly (styrene sulfonic acid) (PEDOT: PSS) or MoO_3_ for hole transportation and the ZnO or TiO_x_ are the most widely used for cathode interfacial modification^7^,^12^,^13^,^14^. A great effort is currently being exerted across a variety of research fields in the quest to achieve high-performance devices, for example, the synthesis of novel polymer donors, interfacial morphology control, optimized device structure and processing optimization. These devices have shown high power conversion efficiencies (PCE) approaching to 10%^4^,^15^,^16^,^17^.

Unlike traditional bulk solar cells prepared from silicon or polysilicon, organic absorbing materials with confined absorption band can selectively be either transparent or semi-transparent in different regions of the solar spectrum^11^,^18^. Accordingly, semi-transparent polymer solar cells (STPSCs), an extension of PSCs which utilize transparent conductive materials as both electrodes, have recently gained much scientific attention and are considered to be the highest priority market for PSCs^9^,^19^. The STPSCs offer an extensive spectrum of applications such as power-generating windows for buildings, foldable curtains, mobile terminals and clothes, etc. To date, although many transparent polymer-based organic solar cells have been reported previously^18^,^20^,^21^,^22^,^23^,^24^, the lack of appropriate device structure and suitable materials to use in interfacial layers and electrodes for PSCs has meant that these devices have exhibited either low efficiency (<5%, single junction) or unsatisfactory average visible transmittance (AVT) and color purity for large-scale commercial applications^10^,^11^.

Similar to graphene, other two-dimensional (2D) nanomaterials have been receiving great attention in recent years mainly due to their complementary electronic properties when compared with graphene^25^,^26^,^27^,^28^. Molybdenum disulfide (MoS_2_) is a 2D material consisting of hexagonal sheets of molybdenum (Mo) sandwiched between two hexagonal sheets of sulfur (S). MoS_2_ exhibits specific anisotropic mechanical and electrical properties due to the weak bonding between layers^29^,^30^. A mobility value of over 200 cm^2^ V^−1^ s^−1^ has been reported in a field-effect transistor architecture^31^,^32^. It has also been demonstrated that MoS_2_ could act as Schottky-barrier active layer or hole transport layer (HTL) in photovoltaic^33^,^34^,^35^,^36^,^37^. However, MoS_2_ has not been taken full advantages of its 2D plane properties, even though there are few traps on the layer surface due to its lack of dangling bonds.

We report here on materials and processes for reliable and cost-efficient processing of ITO-free semi-transparent polymer solar cells by introducing ultra-thin versatile MoS_2_ nanosheets as interfacial layers and semi-transparent electrodes with employing a silver nanowires–n-type MoS_2_ nanosheets (AgNW-MoS_2_ NSs) cathode and thin Ag metal anode. This is the first time to report a novel AgNW-MoS_2_ NSs/n-MoS_2_ NSs composite cathode can form excellent 2D morphology for interfacial contact and optical distribution in devices, and p-MoS_2_ NSs/ thin Ag metal is found to achieve a suitable anode with high conductivity and transparency at the same time. The ultra-thin versatile MoS_2_ can effectively avoid aggregation of AgNW electrode and is beneficial for interfacial modification of devices, proposing the MoS_2_ can replace the traditional electrode and interface layer in organic optoelectronic materials. Incorporation of the AgNW-MoS_2_ NSs/n-MoS_2_ NSs cathode and p-MoS_2_ NSs/ thin Ag as the hybrid anode not only enhances fill factor of devices but also improves the light absorption in the BHJ layer and the transmittance of the devices simultaneously as shown in Fig. 1. After optimization of the devices, combination of material selection and device engineering facilitates the fabrication of ITO-free PSCs and STPSCs with good average performance of 8.00% (best value of 8.72%) and 6.02% (best value of 6.55%), respectively, based on poly[4,8-bis-(2-ethyl-hexyl-thiophene-5-yl)-benzo[1,2-b:4,5-b]dithiophene-2,6-diyl]-alt-[2-(2-ethyl-hexanoyl)-thieno[3,4-b]thiophen-4,6-diyl] (PBDTTT-C-T)^38^. (6,6)-phenyl-C_70_ butyric acid methyl ester (PC_70_BM) (PBDTTT-C-T:PC_70_BM) system. Furthermore, these good chrominance large-scale STPSCs module based on the novel device structure can light a LED chip under natural light illuminated, which shows great application possibility in power-generating windows for building integrated photovoltaic (BIPV).

## Methods

### Sample Preparation

Chemically exfoliated Molybdenum sulfide (MoS_2_) nanosheets (NSs) were synthesized through Li intercalation. 0.8 g raw MoS_2_ powders (Sigma-Aldrich) were immersed in 24 mL n-butyllithium (n-BuLi, 2.5 M hexane solution) solution. The mixture was stirred at room temperature for 48 h under an Ar atmosphere and then allowed to settle for hours. The mixture was centrifuged several times to remove LiOH and unexfoliated material. The obtained MoS_2_ NSs were n-type materials, for the p-type MoS_2_ NSs were then prepared after 30 min oxygen plasma treatment (PDC-32G-2 PLASMA CLEANERM, high level, 18W). The sample was diluted with isopropyl alcohol to ~0.5 mg mL^−1^ for device fabrication.

For the post-treatments, the AgNWs-MoS_2_ ink was manufactured by blending the Ag NWs dispersion (diam. × L 60 nm × 10 μm, 0.5% isopropyl alcohol suspension Sigma-Aldrich) and n-type MoS_2_ isopropyl alcohol solution (1:1,V:V). Preparation of the Ag NWs and AgNWs-MoS_2_ Films: The Ag NW films were prepared using the spin-coating process and were formed on precleaned glass substrates that were attached to a supporting glass substrate. The as-received dispersion containing Ag NWs and AgNWs-MoS_2_ were spin coated for 40 s at speeds ranging from 500–4000 rpm. The dispersion was sonicated for 30 min and shaken well before being the spin-coating process. The formed electrode films were annealed at ≈120 ^o^C for 10 min in a glove box filled with nitrogen (the detail process was shown in support information in Supplementary Fig. S1).

### Device Fabrication

The polymer solar cells were fabricated on Glass/ITO, Glass/AgNW or Glass/Ag NW-MoS_2_ electrodes. For the electron transport layer, the sol-gel derived ZnO film using the reported method^39^ or 40 nm thickness of n-type MoS_2_ was placed on the top of electrode. The thickness of ZnO film is approximately 30 nm, determined by a profilometer (Alpha-Step-IQ). Subsequently, the modified samples were transferred to the nitrogen-filled glove-box. The D-A copolymer-containing photoactive layer was prepared by spin coating (900 rpm) the dichlorobenzene solution of D-A copolymer (PBDTTT-C-T) and PC_70_BM (1:1.5 w/w, polymer concentration of 10 mg mL^−1^) with 3% volume ratio of DIO additive on the modified electrode. The thickness of the photoactive layer was about 100 nm. Then, the MoO_3_ precursor or p-type MoS_2_ solution with optimal thickness was spin-cast on top of the polymer:fullerene composite layer. Finally, the device was pumped down in vacuum (<10^−7^ torr; 1 torr~133 Pa), and a~100 or 30 nm thick Ag electrode was deposited on top. The deposited Ag electrode area defined the active area of the devices as 0.06 or 0.65 cm^2^ (all the areas were tested with an aperture).

### Power-generating glass Fabrication

The power-generating glass was fabricated from eight 0.65 cm^2^ (effective area) **Device E**. All the devices were weld together, and then encapsulating by UV curing glue. The LED chip was accessed in the circuit.

### Characterization

X-ray diffraction (XRD) patterns of the MoS_2_ nanoparticles were carried out on a Bruker D8 Focus X-ray diffractometer operating at 30 kV and 20 mA with a copper target (λ = 1.54 Å) and at a scanning rate of 1 o/min. Sheet resistances of electrodes were measured by using a four point probe setup with a source measurement unit (Keithley 2400). Current-voltage (*J*-*V*) characteristics were characterized using Keithley 2400. The currents were measured in the dark and under 100 mW·cm^−2^ simulated AM 1.5 G irradiation (Abet Solar Simulator Sun2000). All the measurements were performed under ambient atmosphere at room temperature. The scan range is form 0 V to 1V, and 6.7 mV for each step. All the *J*–*V* curves are based on 150 points. The incident photo-to-electron conversion efficiency spectrum (IPCE) were detected under monochromatic illumination (Oriel Cornerstone 260 1/4 m monochromator equipped with Oriel 70613NS QTH lamp), and the calibration of the incident light was performed with a monocrystalline silicon diode. Transmittance spectra were analyzed by UV-vis spectroscopy (Perkin Elmer Lambda 750). The morphologies of films were investigated by atomic force microscopy (AFM) using a Digital Instrumental Nanoscope 31 operated in the tapping mode. The thicknesses of all the layers were measured by surface profilometer (Alpha-Step-IQ). XPS studies were performed on a Thermo-VG Scientific ESCALAB 250 photoelectron spectrometer using a monochromated AlKa (1,486.6 eV) X-ray source. All recorded peaks were corrected for electrostatic effects by setting the C−C component of the C 1s peak to 284.8 eV. The base pressure in the XPS analysis chamber was 2 × 10^−9^ mbar. For the UPS measurements, He I (21.22 eV) radiation line from a discharge lamp was used, with an experimental resolution of 0.15 eV. All the UPS measurements of the onset of photoemission for determining the work function were done using standard procedures with a −5 V bias applied to the sample. Raman spectroscopy was performed using an InVia Raman Microscope system (Renishaw, Inc.), with an Ar+ ion laser operating at 613 nm and 1.2 mW. All the simulation were calculated via Matlab program, the Matlab files were provided from McGehee group, Center for Advanced Molecular Photovoltaics, Stanford University. For the evaluation of the color rendering indices (CRIs), the experimental transmission of each device is folded with the AM1.5 spectrum to obtain the perceived transmission under solar illumination. The resulting data is coupled with the CIE 1931 2^o^ standard observer color matching functions to obtain the corresponding xyY points.

## Results

### Properties of ultrathin MoS_2_ NSs

In order to obtain ultrathin MoS_2_ NSs, the raw MoS_2_ powders were exfoliated by chemical lithium intercalation–exfoliation method^28^,^40^. Generally, the synthetic procedure involves two steps: n-BuLi can intercalate bulk MoS_2_ with lithium atoms and form LixMoS_2_ (0 < x < 1) in the gram scale or more, at room temperature. And then LixMoS_2_ can react with H_2_O rapidly and release large amounts of hydrogen gas, which can push the MoS_2_ layers and cause them to separate from each other and form homogeneous ultrathin MoS_2_ NSs. The powder X-ray diffraction (XRD) patterns of raw and exfoliated MoS_2_ nanosheets (Supplementary Fig. S2, Supporting Information), after chemical exfoliation, show that only the peaks of (002) and (103) plane remain which confirms that the MoS2 nanosheets were successfully striped. And the disappearance of other peaks could proves ultrathin MoS_2_ nanosheets tightly lie on the substrate with preferred orientation^35^. The morphology and structure of the raw MoS_2_ and exfoliated MoS_2_ nanosheets were studied by Transmission electron microscope (TEM), High Resolution Transmission Electron Microscopy (HRTEM), and scanning electron microscope (SEM) characterizations. As shown in Supplementary Fig. S3a-d and S4, bending and folding of the material can be clearly observed from the basal plane of the MoS_2_ NSs, which indicates that the MoS_2_ NSs are very thin. The HRTEM image Supplementary Fig. S2e shows the typical hexagonal single crystal structure of MoS_2_ with a distance of 0.278 nm for the (100) Mo atoms^41^. The selected area electron diffraction (SAED) pattern in (Supplementary Fig. S3f) implies that the MoS_2_ NSs have a single crystal structure with hexagonal symmetry^42^.

**Figure 2:**
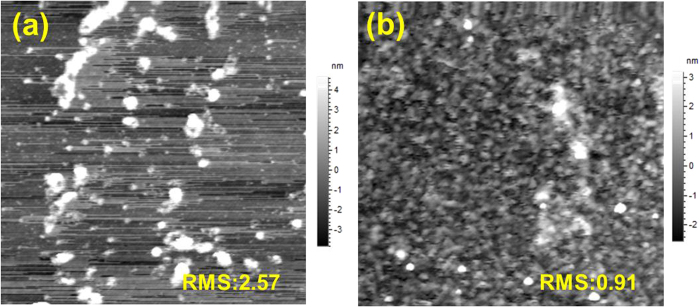
Tapping-mode atomic force microscopy (AFM) images of films (a) raw MoS_2_ film height image, (**b**) exfoliated MoS_2_ NSs film height image, (scan range: 5 μm×5 μm).

Supplementary Fig. S5 exhibits the transmission of the raw MoS_2_ and exfoliated MoS_2_ nanosheets films which were dispersed in isopropyl alcohol (IPA). In the range of the solar spectrum, the raw MoS_2_ and exfoliated MoS_2_ nanosheets films are transparent with relatively high transmission (>80%). The transmission of exfoliated MoS_2_ nanosheets film is slightly higher than the raw MoS_2_ one, probably due to the thinner film of exfoliated MoS_2_ nanosheets layer. In addtion, Fig. 2 demonstrates a quite difference of 2D morphology bettween raw MoS_2_ and exfoliated MoS_2_ nanosheets films. The exfoliated MoS_2_ nanosheets exhibit a much more smooth and homogenous morphology (Rms: 0.91) than raw MoS_2_ film (Rms: 2.57), which could enhance the interfacial contact and favor the better morphology, resulting in improved performance for organic optoelectronic devices^12^,^13^.

### The electric and optical properties of AgNW-MoS_2_ electrodes

**Figure 3:**
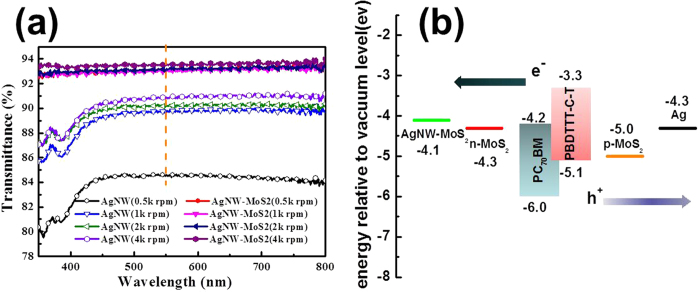
The optical property of AgNW composite electrodes and energy level alignment of devices. (**a**) Transmittance of spin-coated AgNW and AgNW-MoS_2_ on glass deposited at different spin speeds. (**b**) the schematic energy diagram of the electrodes and interfacial layers involved in the STPSCs.

The MoS_2_ NSs were firstly introduced to adding in AgNW ink for transparent electrode. After optimization of the MoS_2_ NSs component in AgNW ink, the optical and electrical properties of the AgNW and AgNW-MoS_2_ electrodes fabricated by different spin speeds are summarized in Table 1 and Fig. 3a and Supplementary Fig. S6. The optical and electrical parameters of pristine AgNW electrode are basically identical with the previous reports. It is well known that a competitively transparent electrode must has a transparency of at least 90% and a sheet resistance of less than 10 Ω/sq^43^, therefore only AgNW ink is hard to satisfy the two requirements at the same time^44^,^45^. Delightfully, the modified AgNW-MoS_2_ loading shows a relative low sheet resistance of 9.8 Ω sq^−1^, and the corresponding transmittance of the electrode also exhibits a high value of 93.1% at 550 nm. It is obvious to found that the transmittance of the electrodes via different speed after the addition of MoS_2_ NSs has a significant increase, mainly attributed to its high refractive index of MoS_2_ NSs and a well 2D plane structure. Moreover, the environmental stability of AgNW and AgNW-MoS_2_ electrodes (500 rpm spin-coating speed) were tested under 50 ^o^C and 70 relative humidity (RH) conditions for 7 days, as shown in Supplementary Fig. S7. AgNW electrode is easy to oxidation under the atmospheric environment^46^,^47^, nevertheless, the incorporation of MoS_2_ NSs in AgNW electrodes show eminent oxidation resistance and moisture absorption stability.

The pristine MoS_2_ NSs are n type semi-conductor materials^34^,^48^, several work has been reported that MoS_2_ could be changed as a p type semi-conductor materials with a relative high work function after UV-ozone plasma treatment^34^,^37^,^48^. Thus, the properties of MoS_2_ NSs before or after the UV-ozone plasma treatment were also investigated. Supplementary Fig. S8 is the X-ray photoelectron spectroscopy (XPS) profiles of n-MoS_2_ NSs (w/o plasma treatment) and p-MoS_2_ NSs (with plasma treatment). The Mo 3d spectra of pristine MoS_2_ NSs exhibit strong Mo^4+^3d_5/2_ and Mo^4+^3d_3/2_ bands at 228.5 eV and 231.8 eV, in agreement with the others work for n-MoS_2_ NSs^35^,^36^. However, the two strong peaks have a notable shift to 235.5 eV and 232.3 eV, respectively, which is consistent with the spectra of MoO_3_^49^. Therefore, it is proved that n-MoS_2_ NSs can be successfully oxidized to p-type materials after plasma treatment^35^.

The work function (*WF*) of the n and p type MoS_2_ NSs was determined via UPS (Supplementary Fig. S9) and Kelvin probe (Supplementary Fig. S10). The *WF* of the n-MoS_2_ NSs (pristine MoS_2_ NSs) is found to be 4.3 eV, while that of the AgNW-MoS_2_ electrode is 4.1 eV, which is even lower than Ag electrode (4.3 eV), meaning more efficient electron collection for cathode^12^. As expected, after plasma treatment, the oxygen doping at the surface of the MoS_2_ NSs film and making the *WF* of p-MoS_2_ NSs upshifts to 5.0 eV, which is nearly to the *WF* of MoO_3_ (5.3 eV). The substantial increased work function is close to the HOMO level of PBDTTT-C-T, as illustrated in Fig. 3b, which can avoid the hole from capturing and gathering at the interface to form a recombination center, leading to more effective hole selection and transportation for PSCs^12^.

### Photovoltaic performance and characterization

**Figure 4:**
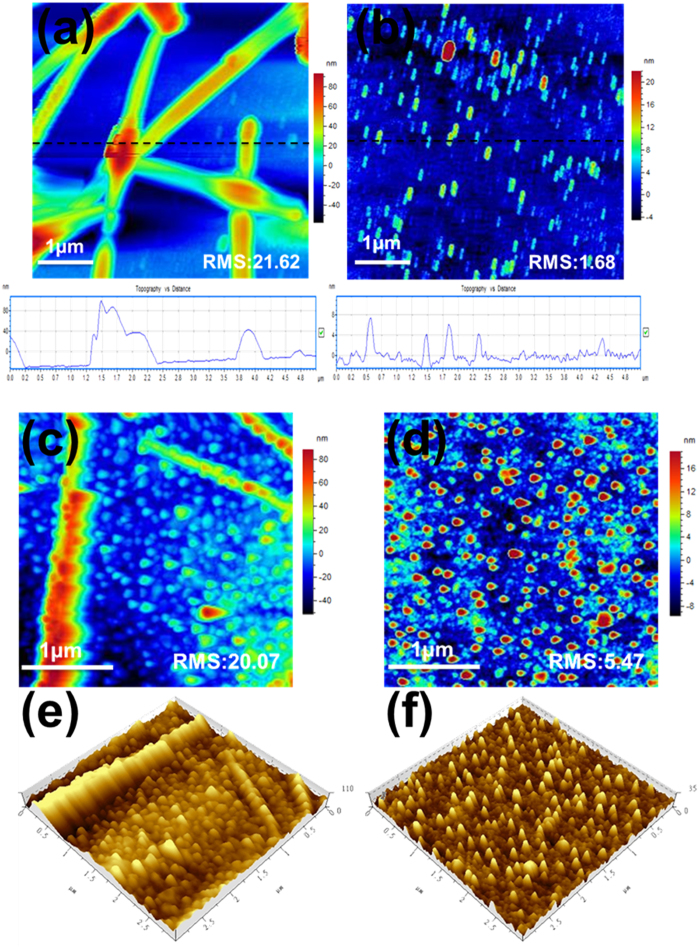
Tapping-mode AFM images of electrodes (**a**) AgNW bottom electrode film height image, (**b**) AgNW- MoS_2_ bottom electrode film height image, (scan range: 5 μm×5 μm) height images of 30 nm thin Ag top electrode film (**c**) which based on BHJ layer (**d**) which based on BHJ/p-MoS_2_ layer. Three-dimensional images of 30 nm thin Ag top electrode film (**e**) which based on BHJ layer (**f**) which based on BHJ/p-MoS_2_ layer, (scan range: 3 μm×3 μm).

Besides suitable energy alignment in the devices, excellent interfacial morphology is also critical for high performance PSCs^13^. The formation of the AgNW and AgNW-MoS_2_ films can also be verified by optical microscope (OM) and AFM images displayed in Supplementary Fig. S11 and Fig. 4a,b. Enormous aggregation of AgNW can be observed from large-scale OM and micro-scale AFM observation, while the AgNW-MoS_2_ film develops more smooth morphology with no apparent aggregation in Supplementary Fig. S11. Intriguingly, the AFM image of AgNW-MoS_2_ film demonstrates highly oriented Ag nanowires arrange uniformly in MoS_2_ NSs background, with a more smooth surface than AgNW one (RMS:1.68 vs 21.62,). These oriented Ag nanowires can provide a convenient pathway for electrons transportation and collection in AgNW-MoS_2_ electrodes, beneficial to the high conductive electrode. In addition, the interpenetrating network structure of AgNW and MoS_2_ NSs also render a great contribution to the homogeneous surface of AgNW-MoS_2_ film and high transmittance. The Ag composite electrodes could also have a great influence on active layer, as shown in Supplementary Fig. S12. Supplementary Fig. S12a shows the morphology of BHJ active layer which based on AgNW electrode with a rough surface (RMS: 6.46). However, the AgNW-MoS_2_ film can provide a 2D substrate for deposition of BHJ active layer with a reduced RMS of 1.21. The different BHJ active layers and p-MoS_2_ NSs have a great effect on the morphology of top Ag electrode as well. The AFM height and three-dimensional images of 30 nm Ag film deposited on bare BHJ layer (Fig. 4c,e), display a horrifically rough surface with a RMS of 20.07, leading to the poor device performance (discuss later). In contrast, the Ag film covered on p-MoS_2_ layer/BHJ exhibits an uniform and smooth surface morphology in Fig. 4d,f, which means p-MoS_2_ NSs can act as a soft mattress and provide an ingenious interfacial contact between BHJ layer and Ag electrode.

**Figure 5:**
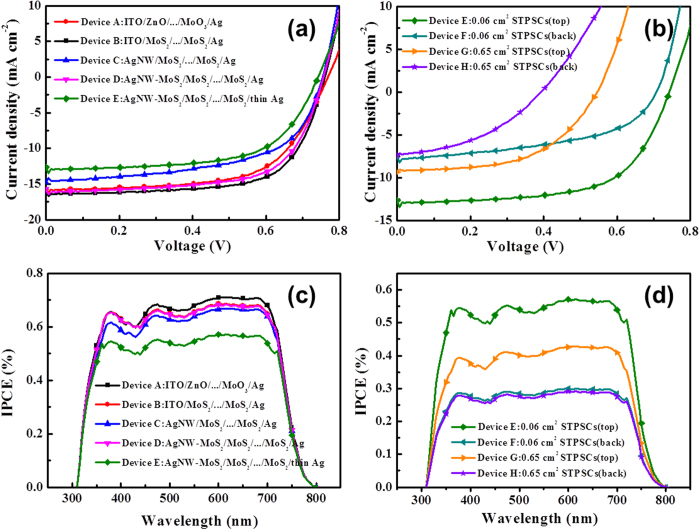
Current (*J*)–voltage (*V*) characteristics and Incident photon-to-current efficiency (IPCE) of cells based on different devices (**a**) *J*–*V* result for Device A-E (**b**) *J*–*V* result for Device E-H, (**c**) IPCE result for Device A-E (**d**) IPCE result for Device E-H.

To determine the function of the versatile MoS_2_ NSs on the optoelectronics, the PSCs based on: (PBDTTT-C-T) :(6,6)-phenyl-C70 butyric acid methyl ester (PC_70_BM), Glass/Cathode/ETL/ PBDTTT-C-T:PC_70_BM/HTL/Ag) were fabricated with the solution-processed 2D n-MoS_2_ NSs films as ETL and solution-processed 2D p-MoS_2_ NSs as HTL. The current density-voltage (*J*–*V*) characteristics of inverted cells with various buffer layers under AM 1.5G irradiation at 100 mW·cm^−2^ are shown in Fig. 5a, and the related electrical parameters are summarized in Table 2. The **Device A** (Glass/ITO/ZnO/BHJ/MoO_3_/Ag(100 nm)) as the control device delivers a PCE of 7.62% with a short-circuit current density (*J*_sc_) of 15.97 mA·cm^−2^, an open circuit voltage (*V*_oc_) of 0.76 V and a fill factor (*FF*) of 0.63. After the PSCs are modified by n-MoS_2_ and p-MoS_2_ interfacial layer, the *FF* of the **Device B** (Glass/ITO/n-MoS2/BHJ/p-MoS2/Ag (100 nm)) increases to 0.70 together with improved PCE and *J*_sc_. Since the *FF* of PSCs is normally determined by the interfacial contact and morphology of the devices, the increase in *FF* can be attributed to the smoother interfacial contact between electrodes and active layer after introducing the 2D MoS_2_ NSs layers. Moreover, the relative high conductive MoS_2_ layers also tend to form favorable interfacial transportation to enhance the charge extraction and reduce the charge combination, leading to the improvement in the *J*_sc_. When the ITO cathode was replaced to AgNW, all of the device parameters, e.g. PCE, *J*_sc_, *V*_oc_ and *FF* are substantially reduced in **Device C** (Glass/AgNW/n-MoS_2_/BHJ/p-MoS_2_/Ag(100 nm)), resulting from the poor transmittance of cathode and reduced light absorption in active layer. Intriguingly, after introducing AgNW-MoS_2_ electrode, the PCE of ITO-free device is further improved from 6.39% for **Device C** to 8.00% for **Device D** (Glass/AgNW-MoS2/n-MoS2/BHJ/p-MoS2/Ag(100 nm)), which is even comparable to the control **Device B** based on ITO cathode. The improved efficiency is mainly related to the increased *J*_sc_ (15.66 mA cm^−2^) and *FF* (0.66), revealing the function of AgNW-MoS_2_ electrode and in good agreement with AFM measurement (Fig. 4 and Supplementary Fig. S12).

To fabricate a semi-transparent polymer solar cells (STPSCs), the top Ag electrode directly decreased Ag thickness from 100 nm to 30 nm^11^, since the control device with 100 nm thick Ag anode was almost opaque (average visible-light transmittance: <0.05). When the thickness of top Ag layer decreases to 30 nm for a thin electrode, the total transmittance of the devices shows an average value of 0.21, as shown in Supplementary Fig. S13. Gratifyingly, the STPSCs based on the structure (**Device E:** Glass/AgNW-MoS_2_/n-MoS_2_/BHJ/p-MoS_2_/Ag(30 nm)) emerges an average PCE of 6.02%, with a *J*_sc_ of 12.66 m^−2^, an *V*_oc_ of 0.75V and a *FF* of 0.64, when the light was illuminated from bottom side, the PCE is the highest values reported so far for ITO-free semi-transparent PSCs devices. Moreover, the PCE of the **Device F** with the light injected from the top Ag side also present a PCE of 2.77%. The effectiveness of **Device E** structure inspires us to apply it for large-scale STPSCs. The *J*–*V* curves of the STPSCs which increase the effective area from 0.06 cm^−2^ to 0.65 cm^−2^ were shown in Fig. 5b and Table 2. the ITO-free inverted device with a structure of Glass/AgNW-MoS_2_/n-MoS_2_/BHJ (0.65 cm^−2^)/p-MoS_2_/Ag (30 nm)) (**Device G**) still shows a PCE as high as 2.71%, with a *V*_oc_ of 0.54 V, a *J*_sc_ of 9.38 mA·cm^−2^, and an *FF* of 0.53, and parameters of the device tested from top Ag side also reach a high level (1.20, **Device H**). The IPCE spectra of different devices based on PBDTTT-C-T:PC_70_BM are shown in Fig. 5c,d, and the values of *J*_sc_ via the IPCE measurement well match with the ones from *I*-*V* curves. These results demonstrate the universality of this novel and large-scale STPSCs.

## Discussion

**Figure 6:**
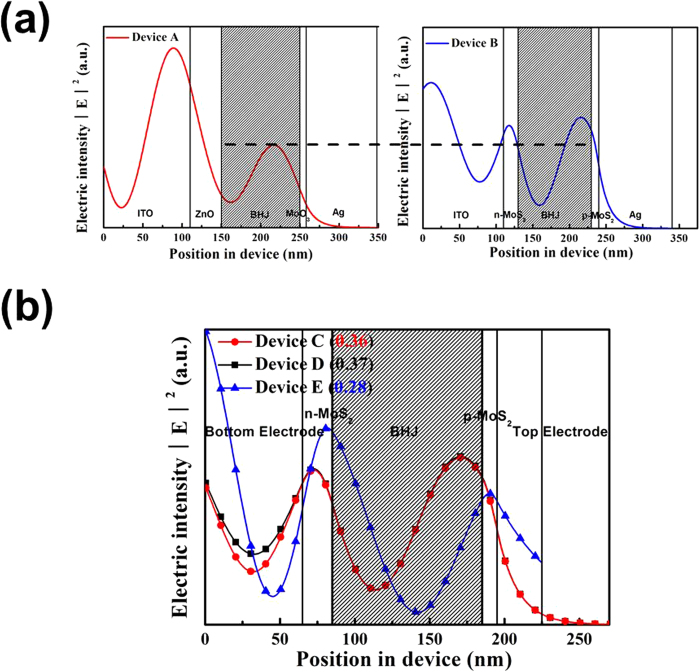
Simulated spatial distribution of the squared optical electric field |E|2 (normalized to the incoming plane wave) for different the devices (**a**) Device A and Device B for 500 nm, (**b**) Device C, Device D and Device E for 400 nm, 500 nm and 600 nm with the same y-axes scale. (The insert figures in brackets mean the relative area ratio in active layer. All the simulation were calculated via Matlab program, the Matlab files were provided from McGehee group, Center for Advanced Molecular Photovoltaics, Stanford University^53^).

To find out the reason for higher efficiency of PSCs caused by introducing the MoS_2_ NSs interfacial layer, the simulation of the spatial distribution of the squared optical electric field (normalized to the incoming plane wave) for the devices with ZnO/MoO_3_ and n-MoS_2_ and p-MoS_2_ were shown in Fig. 6a. The increased *J*_sc_ of these devices based on MoS_2_ interfacial layers can be attributed to high refractive index of n-MoS_2_ NSs (3.25 from 300 nm to 800 nm) leading to the higher light intensity within the active layer, as shown in Fig. 6a. From the figure we also can see that there is a dramatic improvement of the optical electric field intensity of 500 nm wavelength in active layer of the devices with n-MoS_2_/p-MoS_2_ with respect to the devices with ZnO/MoO_3_. Furthermore, the simulation of the spatial distribution of the squared optical electric field for the **Device C**, **D** and **E** were presented in Fig. 6b, where the figures of 0.36, 0.37 and 0.28 in brackets mean the relative optical electric field intensity of active layer for different devices. It is obviously found that there is no big difference between **Device C** and **D** (0.36 vs 0.37, the relative area ratio). The thickness of the top Ag electrode sharply decreases from 100 nm to 30 nm, while the relative optical electric field intensity of active layer slightly reduces to 0.28 for **Device E**. In addition, to gain deeper insight into the performance of the large-scale STPSCs were illuminated under different light intensity, we studied a power law dependence of *J*_*sc*_ upon illumination intensity is generally observed in PSCs and can be demonstrated as:


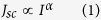


where *I* is the light intensity and α is the exponential factor^50^,^51^. In Supplementary Fig. S14, the data are plotted on a log−log scale and fitted to a power law using eq 1: α = 0.955, higher than the reference device^52^, indicating that bimolecular recombination could be less in the cell with the MoS_2_ NSs transport layer. It is also found that the STPSCs based on **Device E** structure behave an excellent performance under weaker illumination, as shown in Supplementary Fig. S14.

**Figure 7:**
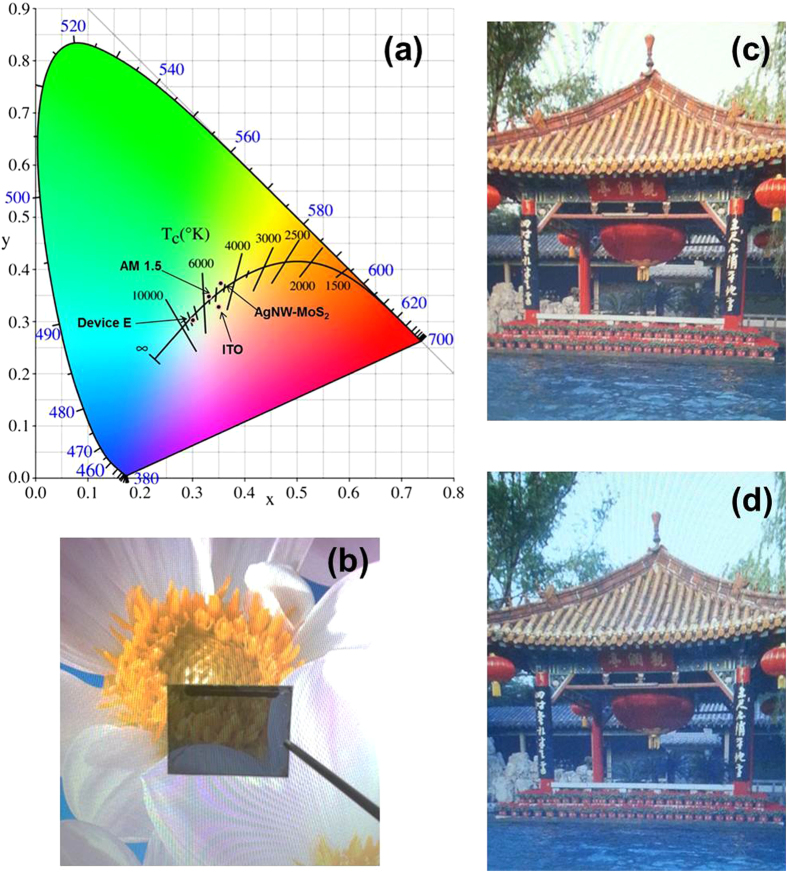
Color properties of the electrode (**a**) The representation of color coordinate of the studied different electrodes and **Device E** on CIE 1931xyY chromaticity diagram. (**b**) the picure of **Device E**. Two digital photographs (**c**) taken through and (**d**) not taken through the STPSCs based on **Device E** structure. (The photographs were taken by Xiaotian Hu).

**Figure 8:**
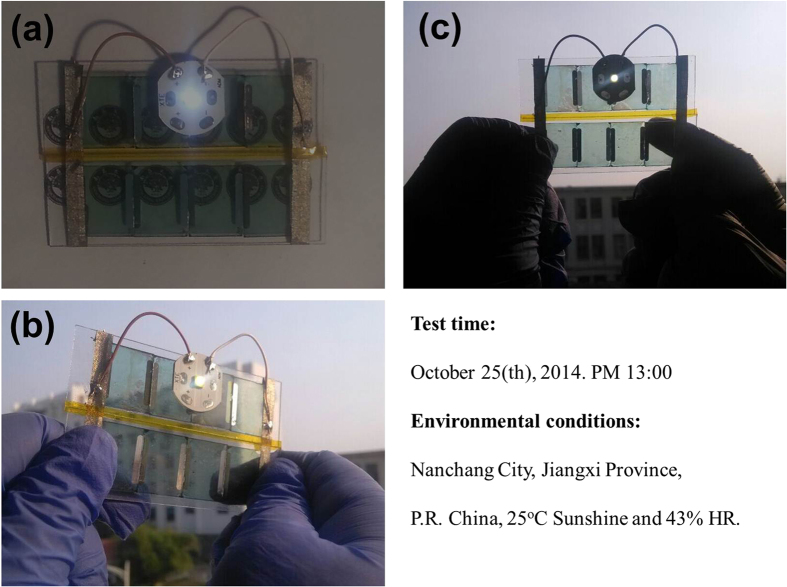
Power-generating glass images. Power-generating glass was fabricated via combine eight 0.65 cm^2^ deivces E, the devices were encapsulated by UV curing glue. To test the power of this semi-transparent solar cell glass, a 5 mW LED chip was put in circuit. (**a**) the power-generating glass was illuminated under 1.5 AM solar condition (**b**) the power-generating glass was tested from back side out of doors (**c**) the power-generating glass was tested from front side out of doors.

To evaluate the transparency perception of the above STPSCs in human eyes, the tristimulus value (X, Y, Z) and the color coordinates (x, y) were calculated from the transmission spectra while the incident light source was replaced with the AM 1.5 spectrum. The color coordinates of the studied STPSCs with different thickness of Ag on CIE 1931 chromaticity diagram are illustrated in Fig. 7a,b. The corresponding coordinates of the ITO, AM 1.5 point, AgNW-MoS_2_ and **Device E** are (0.350, 0.326), (0.359, 0.355), (0.333, 0.347), and (0.313, 0.329), respectively. All these films show very nice transparency color perception that is close to AM 1.5 point. Fig. 7c,d show two digital photographs taken through and not taken through the STPSCs based on **Device E** structure. There is no big aberration between the two pictures, which means it could provide an opportunity for these STPSCs applying in power-generating windows. In order to further to prove the possibility, a power-generating glass was fabricated which is made up of eight 0.65 cm^2^ **Devices E**, as shown in Fig. 8. Fig. 8a delivers the power-generating glass illuminated under 1.5 AM solar condition and the LED chip basically can achieve the rated power. Miraculously, the solar cell glass presents an excellent performance out of doors condition (Test time: October 25^th^, 2014 PM 13:00 and Environmental conditions: Nanchang City, Jiangxi Province, P.R China, 25 ^o^C Sunshine and 43% HR). The photocurrent of power-generating glass is enough to light the LED chip regardless of illumination direction from the front or back side. From these applied tests, it can be sure to say, this kind of power-generating glass provide a great opportunity for building integrated PV (BIPV).

## Conclusions

In summary, we have demonstrated a versatile MoS_2_ nanosheets with well 2D structure that were introduced into Ag nanowires transparent electrode, anode and cathode interfacial layers in PSCs. High performance ITO-free PSCs based on Glass/AgNW-MoS_2_/n-MoS_2_/BHJ/p-MoS_2_/Ag(100 nm) achieve a high PCE of 8.00%.

Furthermore, a novel STPSC is incorporating the MoS_2_ NSs in interfacial layers and AgNW transparent electrode with admirable interfacial contact and superior light-harvesting abilities. The STPSCs exhibited good PCE and AVT with neutral color perception close to that of standard sunlight, which is the highest performance for ITO-free semi-transparent PSCs devices reported up to now. The delightful results demonstrate STPSCs do have great values in power-generating windows of building integrated PV in near future.

## Additional Information

**How to cite this article**: Hu, X. *et al.* Versatile MoS_2_ Nanosheets in ITO-Free and Semi-transparent Polymer Power-generating Glass. *Sci. Rep.* **5**, 12161; doi: 10.1038/srep12161 (2015).

## Supplementary Material

Supplementary Information

## Figures and Tables

**Figure 1:**
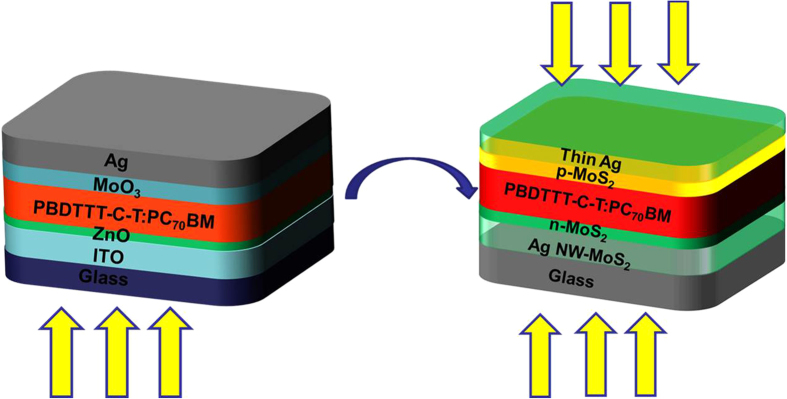
Device configuration of common inverted PSC and MoS_2_ nanosheets (NSs) modified ITO-free STPSC.

**Table 1:** Summary of the key parameters of the AgNW and AgNW-MoS_2_ electrodes fabricated at different spin speeds.

Electrode	Spin speed (rpm)	Thickness (nm)	Sheet resistance (Ω sq^−1^)	Transmittance (%, at 550 nm)
AgNW	500	85 ± 8	9.7 ± 0.6	84.5
	1000	61 ± 8	13.5 ± 0.5	89.6
	2000	37 ± 5	19.3 ± 1.7	90.1
	4000	25 ± 5	32.8 ± 2.1	90.8
AgNW-MoS_2_	500	71 ± 8	9.8 ± 0.5	93.1
	1000	58 ± 7	10.4 ± 0.5	93.0
	2000	32 ± 5	17.3 ± 1.1	93.1
	4000	22 ± 5	28.6 ± 2.2	93.4

**Table 2:** Performance of PSCs (PBDTTT-C-T:PC_70_BM) with different devices under the illumination of AM1.5G, 100 mW/cm^2^.

Device (BHJ:PBDTTT-C-T:PC_71_BM)	*J*_sc_ (mA cm^−2^)	*V*_oc_ (V)	*FF*	PCE (%)	Area (cm^−2^)	AVT^d^ (%)
**A** Glass/ITO/ZnO/BHJ/MoO_3_/Ag(100 nm)	15.79 ± 0.51	0.76 ± 0.01	0.63 ± 0.03	7.62 ± 0.59(8.21)^a^	0.06	<0.05
**B** Glass/ITO/n-MoS_2_/BHJ/p-MoS_2_/Ag(100 m)	15.96 ± 0.52	0.76 ± 0.01	0.70 ± 0.02	8.43 ± 0.62(9.05)^a^	0.06	<0.05
**C** Glass/AgNW/n-MoS_2_/BHJ/p-MoS_2_/Ag(100 nm)	14.79 ± 0.55	0.75 ± 0.01	0.57 ± 0.03	6.39 ± 0.49(6.88)^a^	0.06	<0.05
**D** Glass/AgNW-MoS_2_/n-MoS_2_/BHJ/p-MoS_2_/Ag(100 nm)	15.6 6± 0.61	0.76 ± 0.01	0.67 ± 0.03	8.0 ± 0.72(8.72)^a^	0.06	<0.05
**E** Glass/AgNW-MoS_2_/n-MoS_2_/BHJ/p-MoS_2_/Ag(30 nm) (0.06 cm^2^)	12.66 ± 0.41	0.75 ± 0.01	0.64 ± 0.03	6.02 ± 0.53(6.55)^a^	0.06	21.18
**F** Glass/AgNW-MoS_2_/n-MoS_2_/BHJ/p-MoS_2_/Ag(30 m) (0.06 cm^2^)	7.54 ± 0.52	0.70 ± 0.01	0.52 ± 0.05	2.77 ± 0.40(3.17)a,^b^	0.06	21.64
**G** Glass/AgNW-MoS_2_/n-MoS_2_/BHJ/p-MoS_2_/Ag(30 nm) (0.65 cm^2^)	9.38 ± 0.48	0.54 ± 0.01	0.53 ± 0.05	2.71 ± 0.42(3.13)^a^	0.65	20.97
**H** Glass/AgNW-MoS_2_/n-MoS_2_/BHJ/p-MoS_2_/Ag(30 nm) (0.65 m^2^)	7.43 ± 0.41	0.39 ± 0.02	0.40 ± 0.07	1.20 ± 0.31(1.51)a,^b^	0.65	21.11

^1^best device PCE.

^2^the devices were tested from the back side (top Ag side).

^3^All values represent averages from 12 devices on a single chip, and the areas were tested with an aperture.

^4^average visible-light transmittance from 300 nm to 800 nm.
