# Supplementary material for: Versatile MoS2 Nanosheets in ITO-Free and Semi-transparent Polymer Power-generating Glass
Source: Sci Rep. 2015 Jul 16;5:12161. doi: 10.1038/srep12161 (PMC4648440; doi:10.1038/srep12161)
Supplement: Supplementary Information [file srep12161-s1.pdf]

Supporting Information

Versatile MoS_2 Nanosheets in ITO-Free and Semi-transparent Polymer Power-generating Glass

Xiaotian Hu, Lie Chen, Licheng Tan, Yong Zhang, Lin Hu, Bing Xie, Yiwang Chen*

X. Hu, Prof. L. Chen, Dr. L. Tan, Y. Zhang, L. Hu, Prof. Y. Chen

School of Materials Science and Engineering/Institute of Polymers, Nanchang University, 999 Xuefu Avenue, Nanchang 330031, China

E-mail: ywchen@ncu.edu.cn (Y. Chen)

Prof. L. Chen, Dr. L. Tan, Prof. Y. Chen

Jiangxi Provincial Key Laboratory of New Energy Chemistry, College of Chemistry, Nanchang University, 999 Xuefu Avenue, Nanchang 330031, China

B. Xie

Center of Analysis and Testing, Nanchang University, 235 Nanjing East Road, Nanchang 330047, China

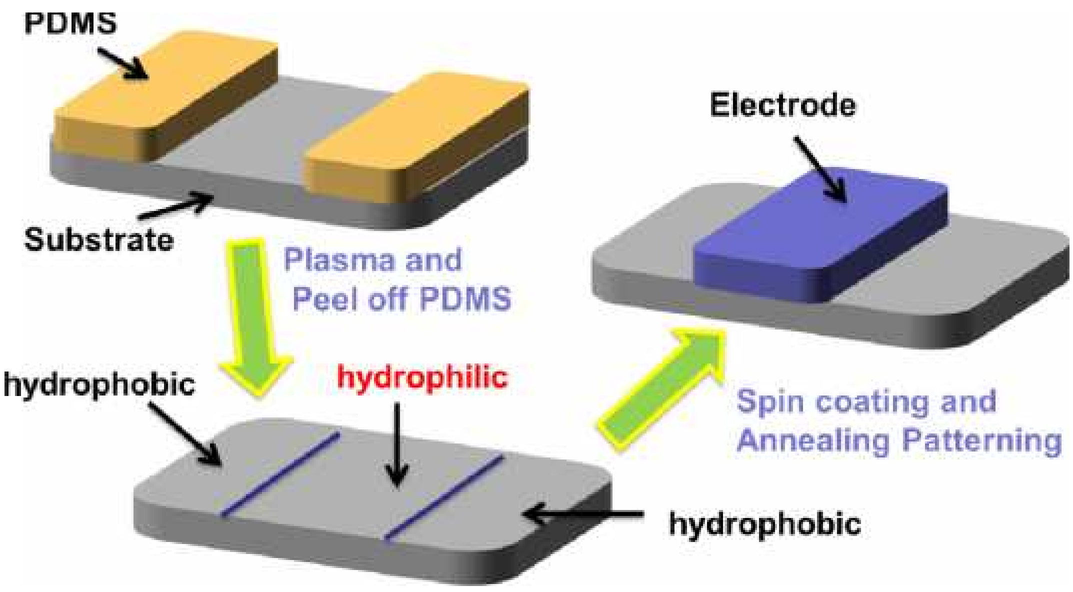

Figure S1 û Fabrication process of ITO-free electrodes. Firstly, PDMS was deposited on half of the PES substrate. After 60 s O_2 plasma treatment the PDMS peeled off, yielding a glass substrate with a half that is hydrophobic (covered by PDMS prior to plasma treatment)

and the other half hydrophilic. Then, conductive ink was spin coated on top the glass substrate and wetted only the hydrophilic part of glass.

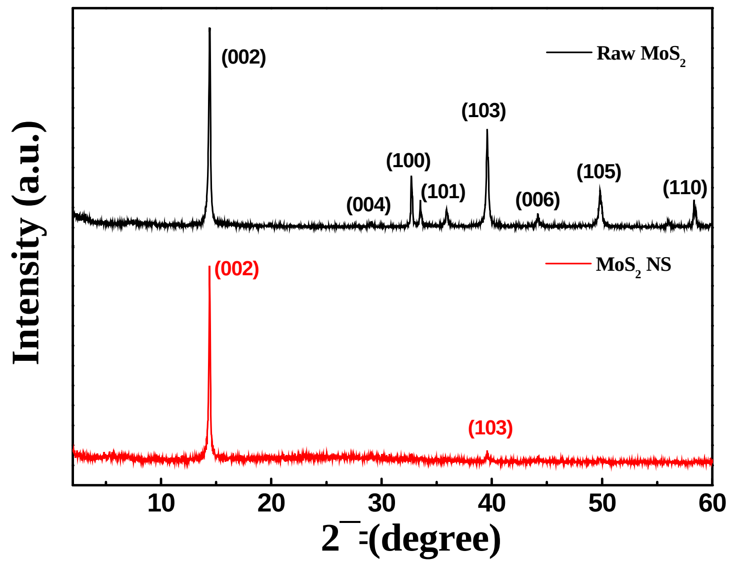

Figure S2 û Powder XRD patterns. The raw MoS_2 and exfoliated MoS_2 NSs deposited on glass substrate.

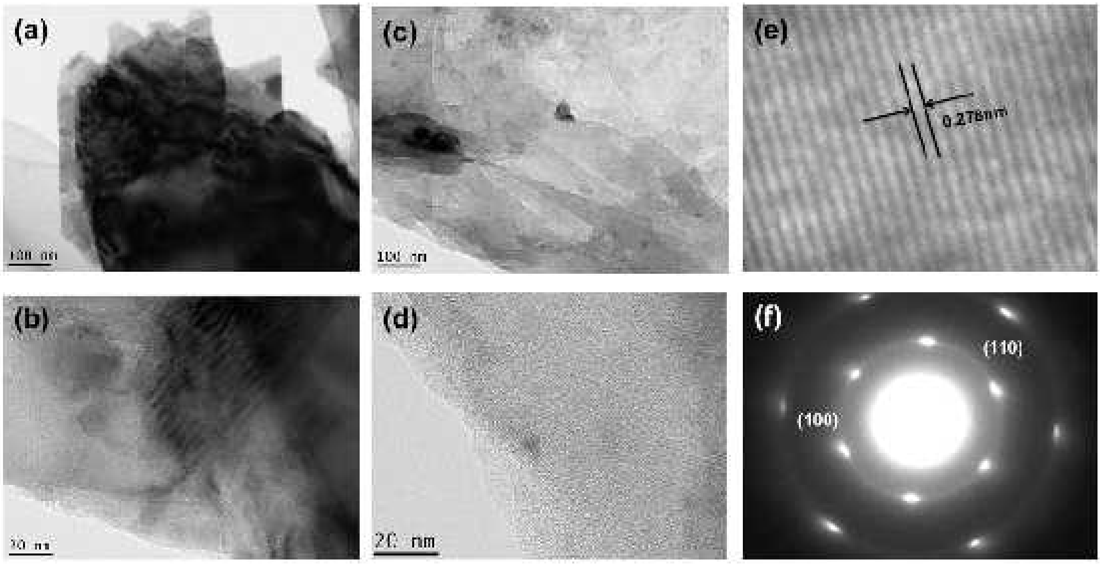

Figure S3 û TEM analysis for MoS_2 powder. (a) and (b) TEM images of raw MoS_2, (c) and (d) TEM images of exfoliated MoS_2 NSs, (e) HRTEM images of exfoliated MoS_2 NSs, (f) selected area electron diffraction (SAED) pattern of the exfoliated MoS_2 NSs.

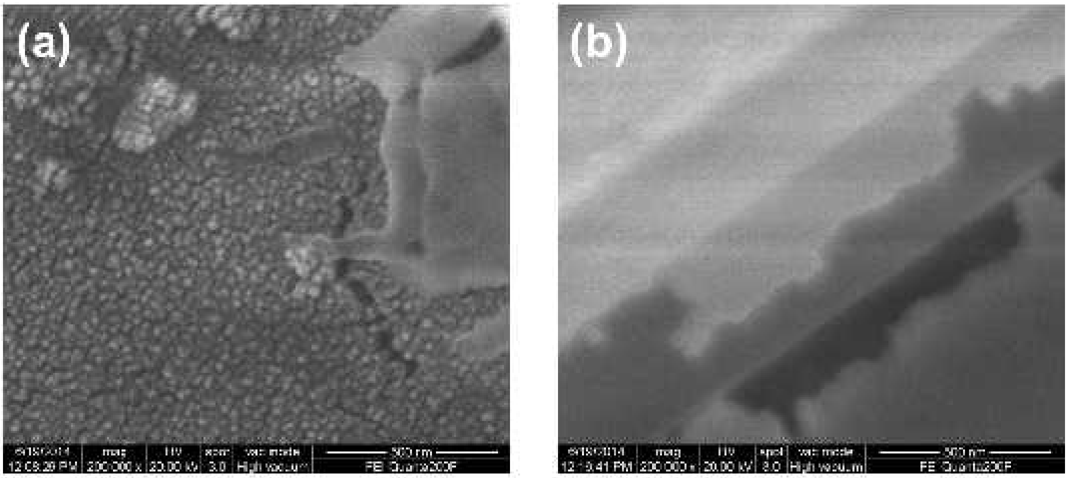

Figure S4 û SEM analysis for MoS_2 powder. The scanning electron microscopy (SEM) images of raw MoS_2 and exfoliated MoS_2 NSs films on glass substrate.

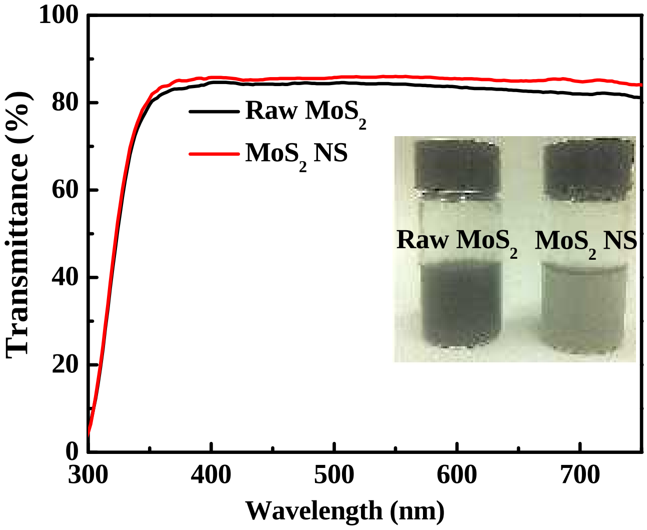

Figure S5 û UV–vis transmittance spectra. Raw MoS_2 and exfoliated MoS_2 NSs films deposited on glass substrate. (inserted images are camera pictures of raw MoS_2 and exfoliated MoS_2 NSs dispersion)

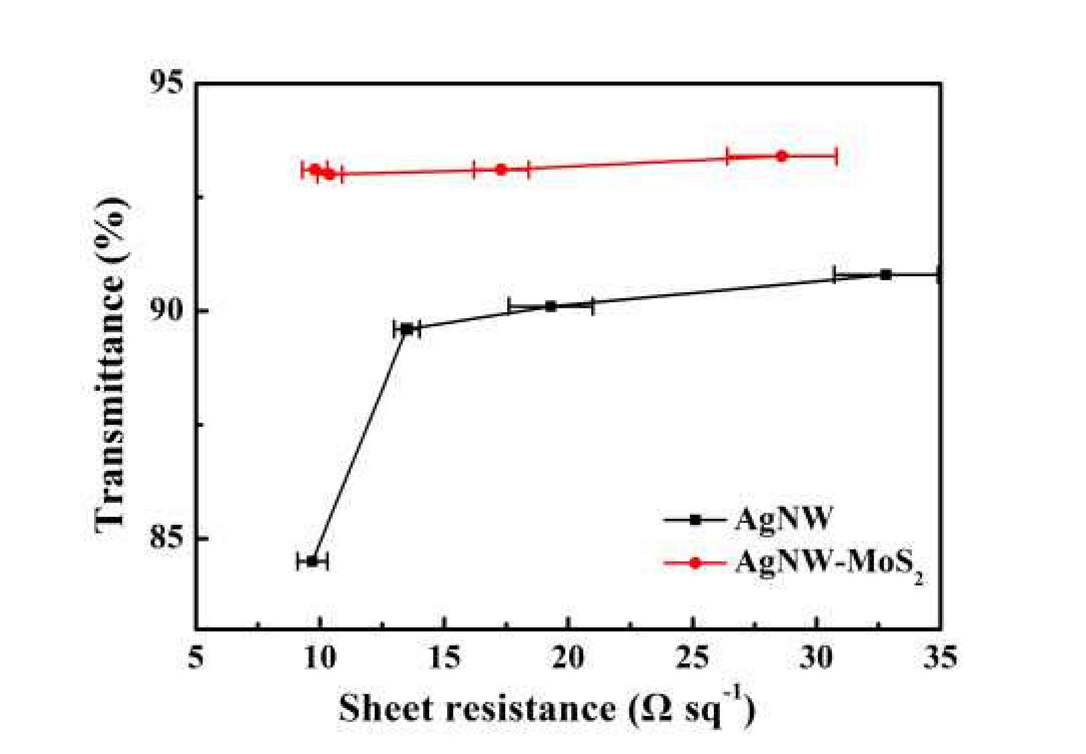

Figure S6 û Electrical and optical properties. The sheet resistance and transmittance of AgNW and AgNW-MoS_2 electrodes.

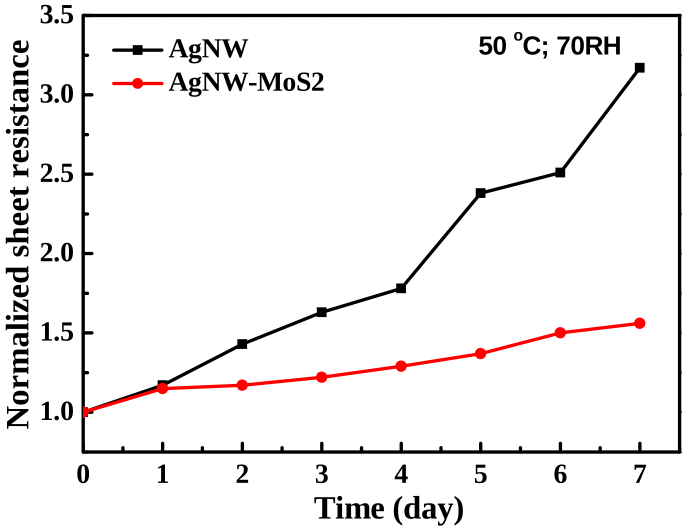

Figure S7 û Hygroscopicity test. Normalized resistance of AgNW and AgNW-MoS_2 electrodes (500 rpm spin-coating speed) were tested under 50 °C and 70 relative humidity (RH) conditions for 7 days.

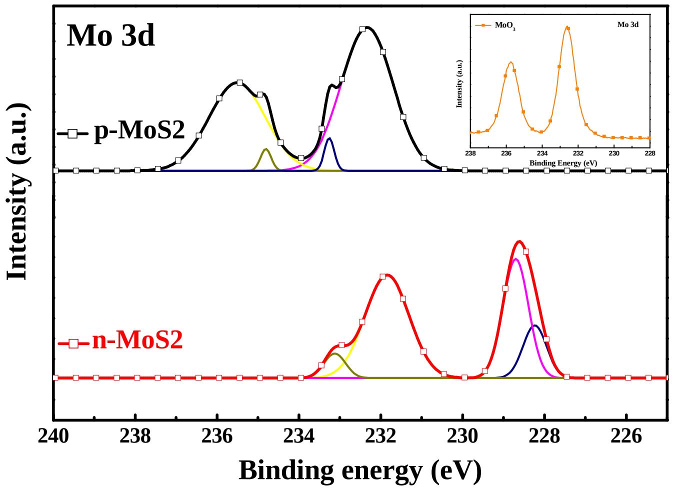

Figure S8 û Composition analysis. Mo 3d region and of X-ray photoelectron spectroscopy (XPS) profiles of n-MoS_2 NSs (w/o plasma treatment) and p-MoS_2 NSs (with plasma treatment). The insert picture is the XPS profiles of 10 nm MoO_3 for comparison.

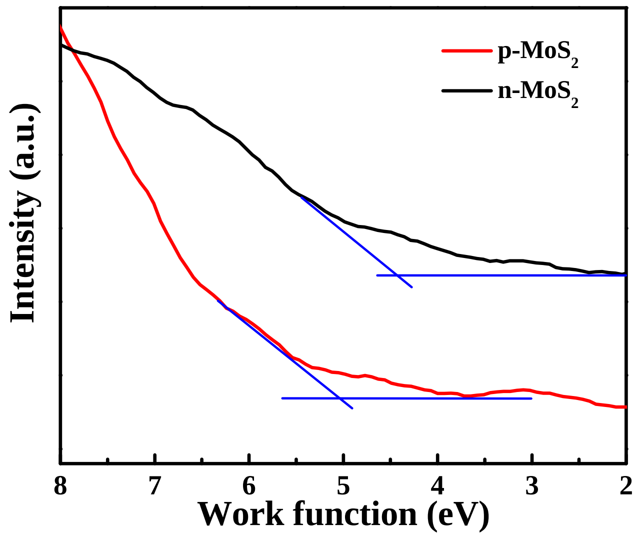

Figure S9 û Work function of p-MoS_2 and n-MoS_2 films measured by ultraviolet photoelectron spectroscopy (UPS).

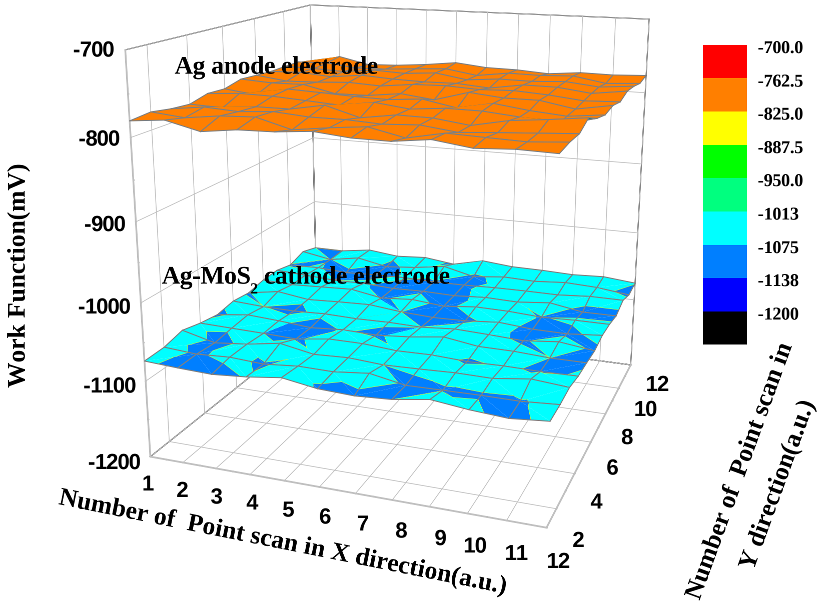

Figure S10 û The work functions of the modified cathode were investigated using a Kelvin probe (KP 6500 Digital Kelvin probe, McAllister Technical Services. Co., Ltd.). The samples were measured in a conditioned chamber where the O_2 level is < 25 ppm. The electronic work functions gap between the samples and standard gold probe shown in **Figure S10.** The actual work function of the samples can be obtained through the equation:

$$WF = WF_0 + \Delta WF$$

Where WF is the sample work function, WF_0 is standard gold probe work function (5.1 ev), and ΔWF is the work function gap between samples and standard gold.

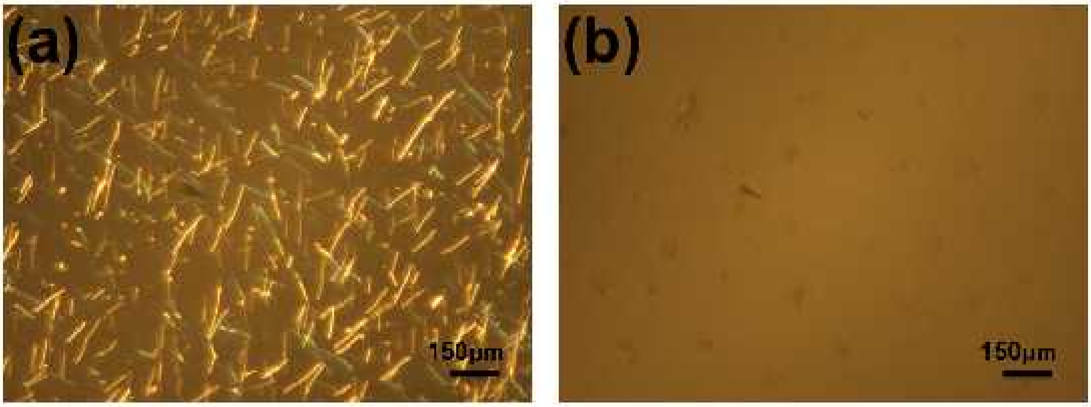

Figure S11 û The optical microscope images of AgNW and AgNW-MoS_2 electrodes.

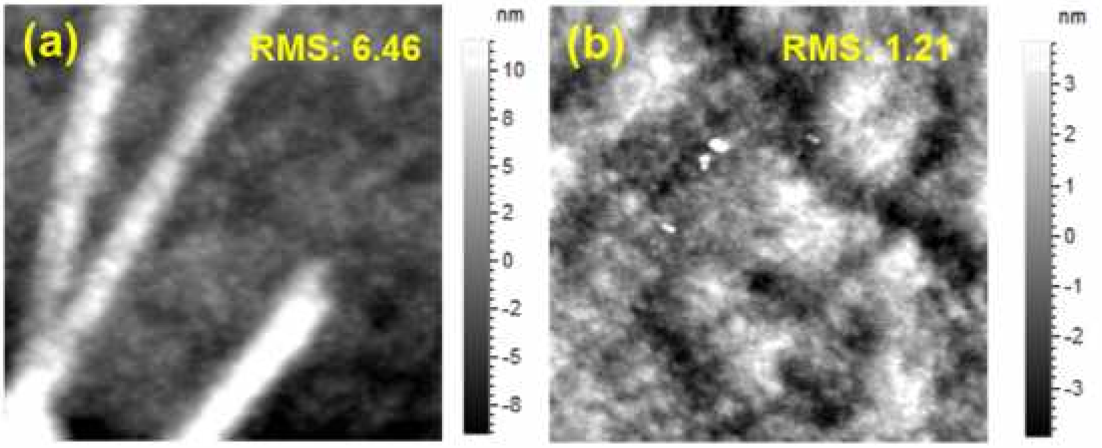

Figure S12 û Tapping-mode AFM height images. (a) BHJ active layer based on AgNW electrode, (b) BHJ active layer based on AgNW-MoS_2 electrode,(scan range:3μm×3μm).

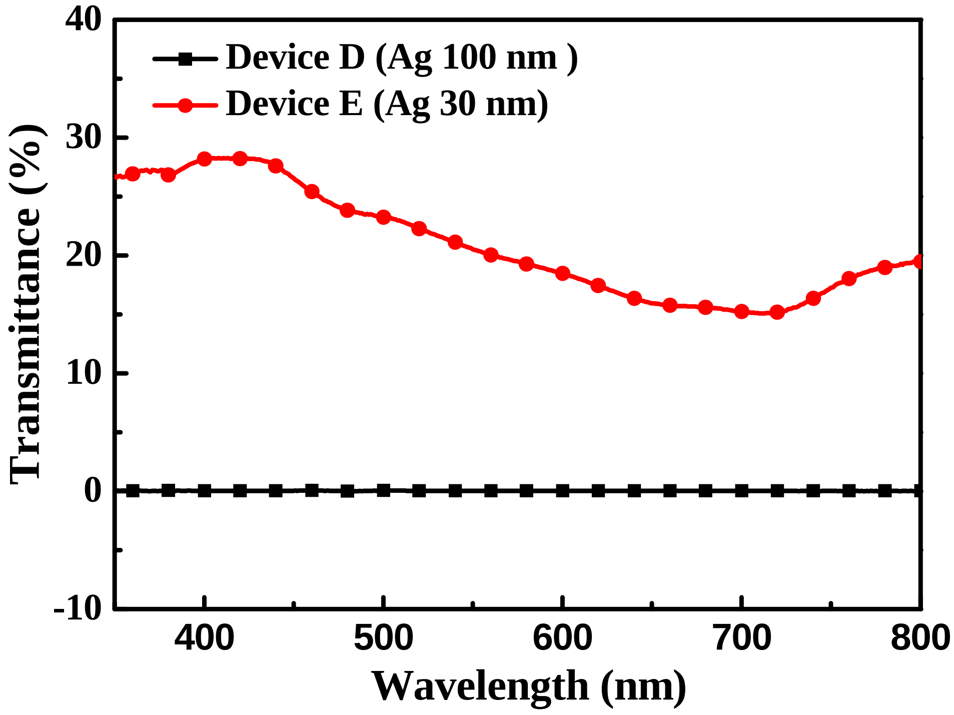

Figure S13 û The transmittance spectra of Device D and E in the visible range.

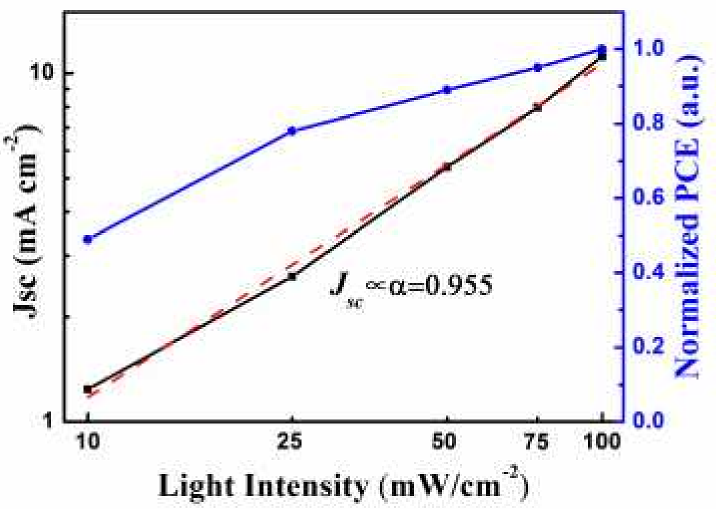

Figure S14 û **Measured J_{SC} of cells based on Device E layer plotted against light intensity (symbols) on a logarithmic scale.** Fitting a power law (solid lines) to these data yields α and normalized PCE under different light intensity.
